# Serine Alleviates Dextran Sulfate Sodium-Induced Colitis and Regulates the Gut Microbiota in Mice

**DOI:** 10.3389/fmicb.2018.03062

**Published:** 2018-12-10

**Authors:** Haiwen Zhang, Rui Hua, Bingxi Zhang, Xiaomeng Zhang, Hui Yang, Xihong Zhou

**Affiliations:** ^1^Key Laboratory of Tropical Animal Breeding and Epidemic Disease Research of Hainan Province, Hainan University, Haikou, China; ^2^Key Laboratory of Tropical Biological Resources of Ministry of Education, Haikou, China; ^3^Key Laboratory of Agro-ecological Processes in Subtropical Region, Institute of Subtropical Agriculture, Chinese Academy of Sciences, Changsha, China

**Keywords:** colitis, inflammation, microbiome, morphology, serine

## Abstract

Serine alleviates inflammatory responses and is beneficial for gut health; however, whether it exerts any effects on ulcerative colitis or regulates intestinal microbiota remains unknown. We investigated the effects of serine supplementation on colonic morphology, inflammation, and microbiota composition in dextran sulfate sodium (DSS)-induced colitis model in mice. Acute colitis was induced through the oral intake of 3.5% DSS in water for 7 days. Mice with acute colitis were divided into two groups; The DSS and Ser-treated groups were rectally administrated with PBS or 1% (w/v) serine (40 mg/kg body weight) for 7 days. The results showed that serine decreased the disease activity index, as well as myeloperoxidase, eosinophil peroxidase, and proinflammatory cytokine concentrations in colonic tissue, while serine improved colonic morphology and inhibited cell apoptosis in colitis mice. In addition, 16S rRNA phylogenetic sequencing revealed a shift in bacterial community composition, and changes in microbiota functional profiles following serine supplementation, although no significant difference in α-diversity analysis was observed. The effects of serine supplementation helped on the recovery of major perturbations to macrobiotic functions, such as amino acids metabolism; tissue replication and repair; and cell growth and death. Serine might have great potential for the renewal of colonic tissue in DSS-induced colitis.

## Introduction

Ulcerative colitis (UC) is one of the major forms of inflammatory bowel disease (IBD) characterized by inflammation of the rectal and colonic mucosa. Although the etiology of UC is unclear, the consensus is that the combined effects of several factors, such as genetic susceptibility factors, impaired intestinal integrity, dysfunctional immune responses and their interactions with intestinal microbiota, as well as environmental factors contribute to the occurrence and development of this disease (Munyaka et al., 2016). In particular, alterations in gut microbiota composition and functions have been demonstrated to play critical roles in human IBD at different stages of the disease (Tamboli et al., 2004; Morgan et al., 2012). Various therapies involving alleviation of inflammation and restoration of the intestinal microbiota have been suggested for the prevention and treatment of UC, including probiotics, prebiotics and dietary supplements such as phytonutrients and propolis (Yeom et al., 2016; Rodriguez-Nogales et al., 2017; Wang et al., 2017). Serine, long considered as a nutritionally non-essential amino acid, has recently been suggested to act as a conditionally functional amino acid. Previous studies have demonstrated that dietary serine helps alleviate triglyceride accumulation and oxidative damage in the liver of several rodent models (Sim et al., 2015; Zhou et al., 2017a, 2018; Cao et al., 2018). Moreover, our recent study showed that dietary serine prevented inflammation in the small intestine and maintained barrier integrity (Zhou et al., 2017b). However, whether serine has any effects on colonic inflammation and gut microbiota is yet to be explored.

Most studies examining the effect of dietary supplements on colitis alleviation have used the most common rodent model involving UC-related erosion and inflammation induced by chemicals such as dextran sulfate sodium (DSS) (Hakansson et al., 2015; Munyaka et al., 2016). Consequently, the present study was conducted to determine the effects of serine on intestinal integrity, inflammation, and microbial dysbiosis in DSS-induced colitis. Furthermore, the related functional pathways were also explored. Our results will provide evidences for the therapeutic potential of serine for regulating dysbiosis in UC.

## Materials and Methods

### Chemicals and Kits

Dextran sulfate sodium salt (DSS) and L-serine were purchased from Sigma-Aldrich, (Shanghai, China). The animal diet was purchase from Research Diets, Inc. (New Brunswick, NJ, United States). Cell death detection kit was purchased from Roche (Shanghai, China). ELISA quantitative kits for the detection of IgA, IgG, IgM, IL-1β, IL-6, TNF-α, myeloperoxidase (MPO), and eosinophil peroxidase (EPO) concentrations were purchased from Cusabio Biotech (Wuhan, Hubei, China^1^). The QIAamp DNA stool MiniKit and Gel Extraction Kit were purchased from Qiagen (Hilden, Germany). The Protein Extract Kit was purchased from Keygen (Nanjing, China). Caspase 3, proliferating cell nuclear antigen (PCNA), mammalian target of rapamycin complex I (mTORC1), and general control non-derepressible 2 (GCN2) antibodies were purchased from Cell Signaling (Beverly, MA, United States). EZ-ECL was purchased from Biological Industries (Cromwell, CT, United States).

### Animal Experiments

Twenty-one 9-week-old male C57BL/6 mice were obtained from SLAC Laboratory Animal Central (Changsha, China) and maintained in plastic cages under standard conditions. Standard pelleted diets (Research Diets, Inc.) were supplied *ad libitum*. Acute colitis was induced through the oral intake of 3.5% DSS (w/v, molecular mass of 6,500–10,000 Da; Sigma-Aldrich) in fresh running water *ad libitum* for 7 days. Mice with acute colitis were divided into two groups; The DSS and Ser-treated groups (*n* = 7) were rectally administrated with PBS or 1% (w/v) serine (40 mg/kg body weight) dissolved in PBS once daily for 7 days. Since previous report indicated that almost all of the amino acids were absorbed or catabolized by the small intestine (Wu, 2009) and our preliminary experiments suggested that oral administration of serine had no significant effects on DSS-induced colitis, we finally supplemented serine via rectal administration which is also widely used for the treatment of DSS-induce colitis in mouse model (Tai et al., 2007; Han et al., 2015). In addition, a Control group (*n* = 7) without acute colitis was also rectally administrated with PBS. Mice were kept inversely for 1 min after administration to prevent leakage from the anus as previously did (Tai et al., 2007; Han et al., 2015). Upon completion of the experiment on day 14, blood was obtained from the retro-orbital sinus. Thereafter, the mice were euthanized by cervical dislocation and the colon and colonic digesta were collected. The experimental protocol was approved by the Protocol Management and Review Committee of Institute of Subtropical Agriculture, Chinese Academy of Sciences and mice were cared for and sacrificed according to the animal care guidelines of Institute of Subtropical Agriculture (Changsha, China).

### Assessment of Disease Activity

The disease activity index (DAI) based on body weight change, stool consistency, rectal bleeding, and overall condition of the animal during the experiment was calculated according to a standard scoring system (Zhang et al., 2015). Scores were recorded using the following criteria: Weight loss: 0, no weight loss; 1, weight loss of 0.1–5% (compared to baseline); 2, 5–10%; and 3, >10%. Stool consistency: 0, well-formed pellets; 2, pasty and semi-formed stools that did not adhere to the anus; and 3, liquid stools that adhered to the anus. Rectal bleeding: 0, no blood; 1, small amount of blood in some stool; 2, blood regularly observed in stool; and 3, blood in all stool.

### Histological Analyses

The colon (3.5–4 cm proximal to the anus) was collected and flushed with chilled PBS. Subsequently, the samples were fixed with 10% buffered formalin and embedded in paraffin. Next, 8-μm sections were obtained and stained with hematoxylin and eosin (HE) (Hu et al., 2017). Finally, colonic morphology was observed under light microscopy and representative pictures were taken. Colonic samples were also fixed in 2.5% glutaraldehyde, then further fixed with osmium tetroxide and dehydrated with graded alcohol. Subsequently, an epon-araldite resin was used to embed the dehydrated samples. Ultrathin sections were obtained and stained with uranyl acetate and lead citrate. Finally, alteration of the mitochondrial structure was observed and representative images were obtained using a Zeiss 902 transmission electron microscope (Zeiss, Thornwood, NY, United States). The histological scoring of colitis was performed according to previous study did (Kitajima et al., 2000).

### Assessment of Apoptosis

Colonic tissues were fixed in 10% formaldehyde and embedded in paraffin and then 5-μm sections were obtained. Apoptosis was detected by TUNEL staining using an *in situ* cell death detection kit (Roche). DAPI mounting solution (Vector, Burlingame, CA, United States) was used for nuclei staining. The results were observed under a light microscope and representative pictures were photographed.

### Measurement of Immunoglobulin, Inflammatory Cytokine, Myeloperoxidase, and Eosinophil Peroxidase Concentrations

Immunoglobulin (Ig)A, IgG, and IgM concentrations in sera and IL-1β, IL-6, TNF-α, MPO, and EPO concentrations in the distal colon (2–3.5 cm proximal to the anus) were determined using ELISA quantitative kits (Cusabio Biotech) according to the manufacturer’s instructions.

### Gut Microbiota Profiling

All content within the colon were pooled and homogenized, and then colonic content DNA was extracted using the QIAamp DNA stool MiniKit (Qiagen). DNA samples were further purified from DSS using lithium chloride as previous study did (Viennois et al., 2013). Briefly, DNA were incubated with 0.1 volume of 8 M LiCl diluted in diethylpyrocarbonate (DEPC)-treated water on ice for 2 h and then centrifuged at 14,000 ×*g* for 30 min at 4°C. The supernatants were discarded and the remaining DNA were dissolved in DEPC-treated water. Then the abovementioned procedure was repeated once more. Bacterial 16S rRNA gene sequences (V3–V4 region) were amplified using specific primers containing a barcode. PCR reactions were performed in a final volume of 50 μL consisting of 12.5 μL of Phusion High-Fidelity PCR Master Mix (New England BioLabs Inc., Beverly, MA, United States), 50 ng of template DNA, 1 μL of each primer, and PCR-grade water. Further experiments were carried out with the 400–450 bp PCR products purified using the Qiagen Gel Extraction Kit (Qiagen). Subsequently, MiSeq Illumina sequencing was performed using the IlluminaHiSeq2500 platform (Illumina Inc., San Diego, CA, United States) and 250 bp paired-end reads were obtained. Next, the paired-end reads were merged using FLASH and then assigned to each sample based on their unique barcodes. High-quality clean tags were clustered into (OTUs) using USEARCH according to the QIIME quality-controlled process based on 97% sequence similarity and representative OTUs were used for further analysis using the Greengenes database with the RDP algorithm. Alpha and beta diversity and principal coordinate analysis (PCoA) were performed with QIIME. OTUs were also used for genome prediction of microbial communities by PICRUSt (Phylogenetic Investigation of Communities by Reconstruction of Unobserved States). Each OTU was used to search the pre-calculated genome content for metagenome prediction. KEGG abundances for each metagenome sample were obtained and KEGG levels 1, 2, and 3 were identified to determine the metagenome response to DSS and serine supplementation.

### Western Blot Analysis

Distal colonic samples (2–3.5 cm proximal to the anus) were ground and lysed using a total protein extract kit (Keygen). Each sample (30 μg protein) was separated by SDS-PAGE and transferred to a nitrocellulose membrane (Wang et al., 2016). The membrane was first incubated with primary antibodies against Caspase 3, PCNA, mTORC1, and GCN2 (Cell Signaling) overnight at 4°C and then incubated with the secondary antibody for 2 h at 25°C. The results were visualized via chemiluminescence with EZ-ECL (Biological Industries).

### Statistical Analysis

All statistical analyses were performed by one-way ANOVA using the general linear model procedures and a mixed procedure (PROCMIXED) of SAS software version 9.2 (SAS Institute Inc., Cary, NC, United States). Data are presented as least squares means ± SEM. Mean values were considered significantly different when *P* < 0.05, and 0.05 < *P* < 0.1 was considered as a tendency.

## Results

### Serine Alleviates Colitis Symptoms

**FIGURE 1:**
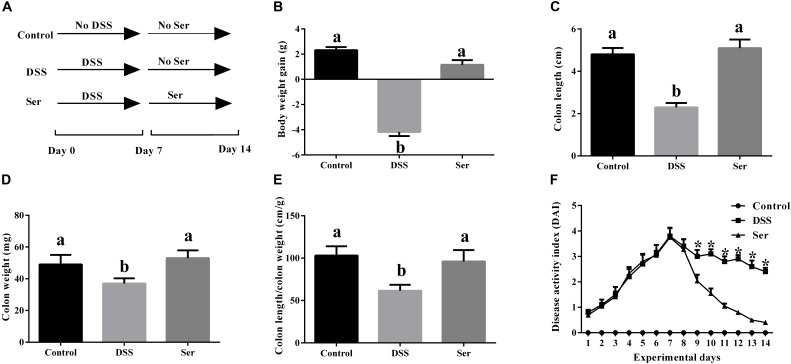
Serine alleviated colitis symptoms. **(A)** A timeline of experiment treatment. **(B)** Body weight gain. **(C)** Colon length. **(D)** Colon weight. **(E)** Colon length/colon weight. **(F)** Disease activity index. Control, mice was rectally administrated with PBS without pretreatment with dextran sulfate sodium; DSS, mice was rectally administrated with PBS after pretreatment with dextran sulfate sodium; Ser, mice was rectally administrated with serine after pretreatment with dextran sulfate sodium. Values are expressed as mean ± SEM, *n* = 7. ^a,b^Means of the bars with different letters were significantly different among groups (*P* < 0.05).

The effects of serine supplementation on colitis symptoms are shown in Figure 1. By day 14, serine supplementation reversed DSS-induced weight loss (Figure 1B). No significant differences in colon length, colon weight, and colon length/colon weight were observed between control mice and serine-supplemented mice (Figures 1C–E). During the experiment, serine supplementation lowered the DAI from day 9 to day 14 (Figure 1F).

### Serine Alleviates DSS-Induced Histopathological Changes and Apoptosis

**FIGURE 2:**
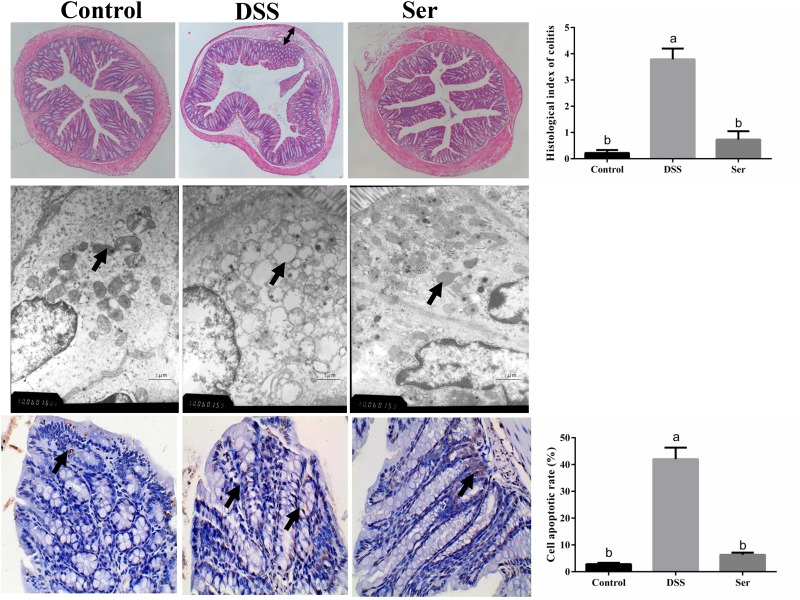
Serine alleviated DSS-induced histopathological changes and apoptosis. (Upper) HE staining of ileum morphology (40×), histological scoring was obtained from the HE results. Arrow, obvious edema. (Middle) ultrastructural observation of colon (transmission electron microscopy, 10,000×). Arrow, mitochondria. (Lower) TUNEL staining (yellow) for assessment of apoptosis (400×), nuclei were stained with DAPI (blue) and cell apoptosis rate was calculated. Control, mice was rectally administrated with PBS without pretreatment with dextran sulfate sodium; DSS, mice was rectally administrated with PBS after pretreatment with dextran sulfate sodium; Ser, mice was rectally administrated with serine after pretreatment with dextran sulfate sodium.

The effects of serine supplementation on DSS-induced histopathological changes and apoptosis are shown in Figure 2. The HE staining results demonstrated pathological changes, such as obvious edema following DSS treatment, in the structure of the mucous layer in the distal colon tissue, while no such changes were observed in serine-supplemented mice. In addition, the TEM results revealed severe mitochondrial edema in the colon tissue of DSS-treated mice. However, serine supplementation alleviated these changes in the mitochondria. Moreover, TUNEL staining showed that the level of apoptosis was higher in DSS-induced mice than in serine-supplemented mice.

### Serine Increases Immunoglobulin Content and Decreases Content of Inflammatory Cytokine and Colonic Infiltration Marker

**FIGURE 3:**
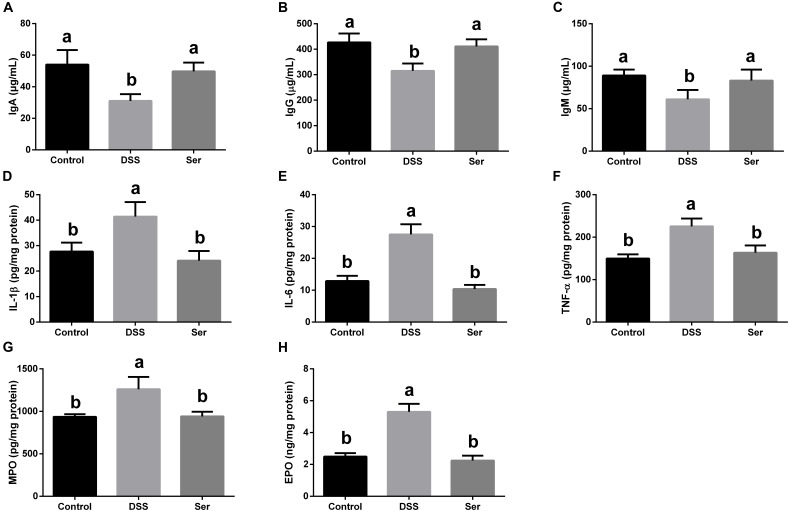
Serine increased content of immunoglobulin and decreased content of inflammatory cytokines and colonic infiltration markers. **(A–C)** IgA, IgG, and IgM concentration in sera. **(D–F)** IL-1β, IL-6, and TNF-α concentration in colonic tissue. **(G,H)** MPO and EPO concentration in colonic tissue. Control, mice was rectally administrated with PBS without pretreatment with dextran sulfate sodium; DSS, mice was rectally administrated with PBS after pretreatment with dextran sulfate sodium; Ser, mice was rectally administrated with serine after pretreatment with dextran sulfate sodium. MPO, myeloperoxidase; EPO, eosinophil peroxidase. Values are expressed as mean ± SEM, *n* = 7. ^a,b^Means of the bars with different letters were significantly different among groups (*P* < 0.05).

Dextran sulfate sodium treatment significantly decreased IgA, IgG, and IgM concentrations (Figures 3A–C) in serum, while increasing IL-1β, IL-6, and TNF-α concentrations in colonic tissue (Figures 3D–F). However, serine supplementation alleviated these DSS-induced changes. In addition, there was a significant increase in MPO and EPO concentrations in the colonic tissue of DSS-treated mice (Figures 3G,H). Supplementing DSS-treated mice with serine significantly decreased MPO and EPO concentrations.

### Serine Alters Colonic Microbiota Composition in Colitis Mice

**FIGURE 4:**
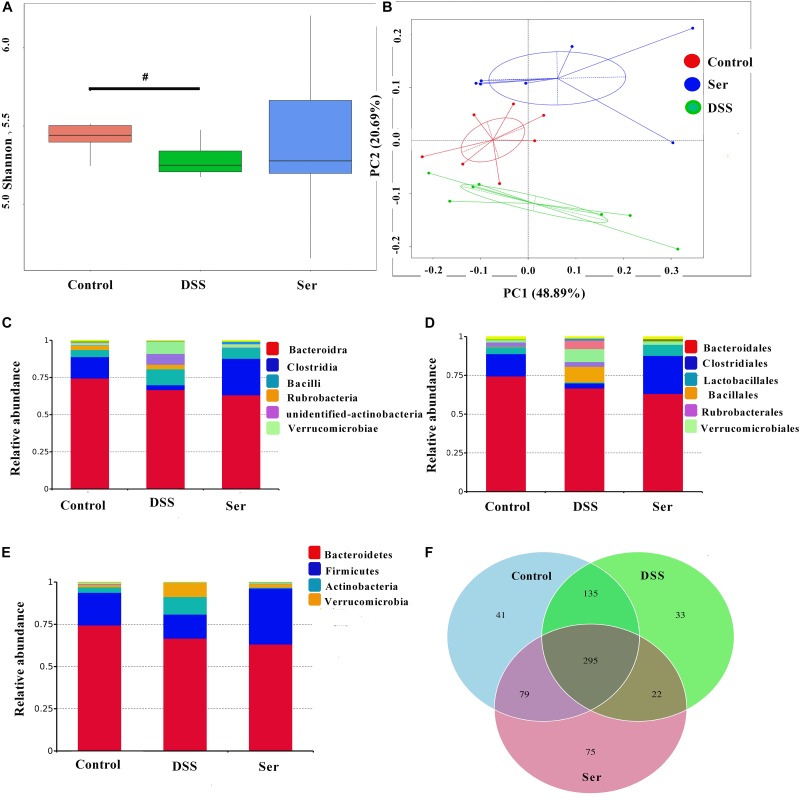
Serine altered the composition of the colonic microbiota in colitis mice. **(A)** Alpha diversity was estimated by the Shannon index. **(B)** PCoA plot of the microbiota based on an unweighted UniFrac metric. Relative abundance of predominant bacteria at the class **(C)**, order **(D)**, and phylum **(E)** level. **(F)** Venn diagram of OTUs. Control, mice was rectally administrated with PBS without pretreatment with dextran sulfate sodium; DSS, mice was rectally administrated with PBS after pretreatment with dextran sulfate sodium; Ser, mice was rectally administrated with serine after pretreatment with dextran sulfate sodium. Values are expressed as mean ± SEM, *n* = 7. ^#^0.05 < *P* < 0.1 was considered as a tendency.

A 16S rRNA phylogenetic approach was used to compare the colonic microbial population of mice from different treatment groups. OTUs were generated from sequences with at least 97% similarity. The alpha diversity of the microbial communities, as indicated by the Shannon index, tended to decrease in DSS-treated mice, while no significant difference was observed between control mice and serine-supplemented mice (Figure 4A). Moreover, we observed a significant difference in beta-diversity among the three treatments based on the weighted PCoA results (Figure 4B). In addition, the results showed that at the class and order levels, *Clostridia* and *Bacteroidia* were the main orders in control mice and serine-supplemented mice, while a decrease of *Clostridiales* was observed in DSS-treated mice (Figures 4C,D). At the phylum level, *Bacteroidetes* and *Firmicutes* were the main phyla (Figure 4E). *Firmicutes* were significantly decreased in DSS-treated mice, while no significant difference was observed between control mice and serine-supplemented mice. The Venn diagram results show that among a total of 680 detected OTUs, 295 OTUs were universal to all samples and there were 75 unique OTUs in serine-supplemented mice (Figure 4F).

### Biofunction Prediction of Microbial Communities

**FIGURE 5:**
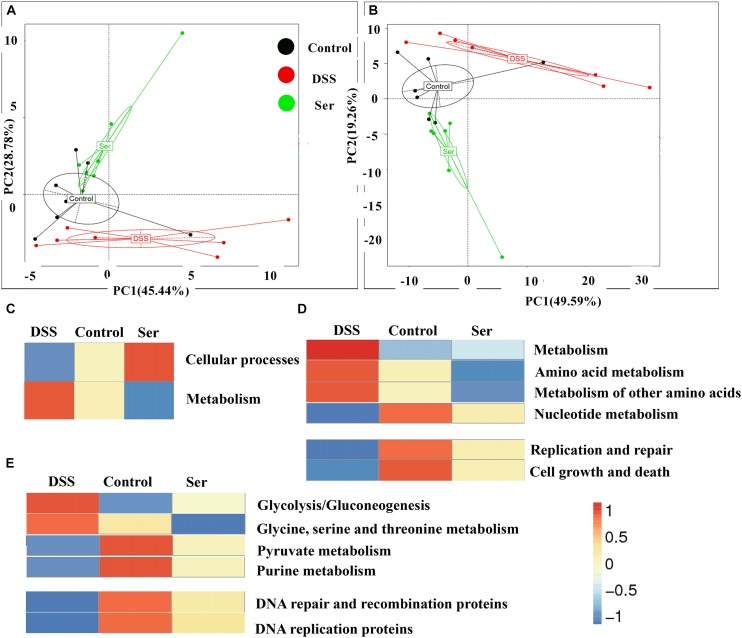
Biofunction prediction of microbial communities. PCoA was generated in KEGG level 2 **(A)** and 3 **(B)**. KEEG pathway annotations in level 1 **(C)**, level 2 **(D)**, and level 3 **(E)**. *n* = 7. Control, mice was rectally administrated with PBS without pretreatment with dextran sulfate sodium; DSS, mice was rectally administrated with PBS after pretreatment with dextran sulfate sodium; Ser, mice was rectally administrated with serine after pretreatment with dextran sulfate sodium.

PICRUSt was performed to predict the functional profiles of the microbial communities. PCoA was generated for different KEGG levels (level 2, Figure 5A; level 3, Figure 5B) to evaluate differences in KEGG abundance among the functional profiles of different treatments. The results showed that the metagenome was greatly modulated in response to DSS treatment and serine supplementation. The most significant KEGG pathway types were cellular processes (predominantly replication and repair, cell growth, and death in level 2) and metabolism (predominantly amino acid and nucleotide metabolism in level 2) gene pathways (Figures 5C,D). These pathways were further analyzed in KEGG level 3. The results showed that serine supplementation alleviated the increased activities of glycolysis/gluconeogenesis and glycine, serine, and threonine metabolism, while improving the decreased activities of pyruvate and purine metabolism, DNA repair and recombination proteins, and DNA replication proteins, which were all induced by DSS treatment (Figure 5E).

### Effects of Serine Supplementation on the Expression of Apoptosis and Proliferation Markers and Proteins Involved in Amino Acid Metabolism

**FIGURE 6:**
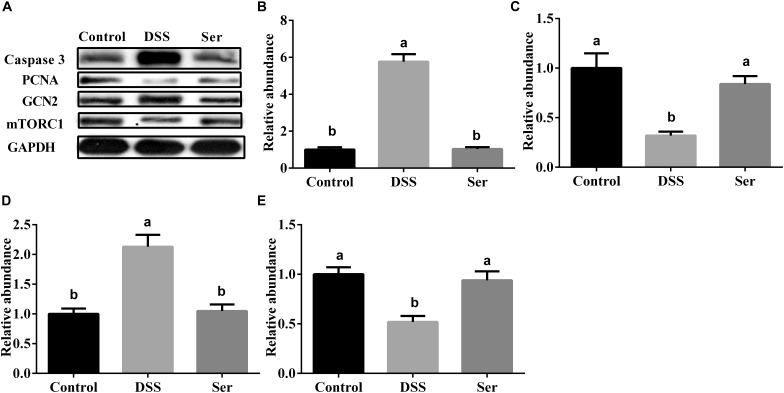
Effects of serine supplementation on expression of markers of apoptosis and proliferation, and proteins involved in amino acid metabolism. **(A)** Western bolting results. **(B)** Relative abundance of Caspase 3. **(C)** Relative abundance of PCNA. **(D)** Relative abundance of GCN2. **(E)** Relative abundance of mTORC1. Each **(B–E)** are the quantification of the WB in **(A)**. Control, mice was rectally administrated with PBS without pretreatment with dextran sulfate sodium; DSS, mice was rectally administrated with PBS after pretreatment with dextran sulfate sodium; Ser, mice was rectally administrated with serine after pretreatment with dextran sulfate sodium. PCNA, proliferating cell nuclear antigen; GCN2, the general control non-derepressible 2; TOR, target of rapamycin. Values are expressed as mean ± SEM, *n* = 3. ^a,b^Means of the bars with different letters were significantly different among groups (*P* < 0.05).

To further explore whether changes in the metabolic pathways of microbial communities affected mucosal renewal and amino acid metabolism in the small intestine, the protein expression of Caspase 3, PCNA, GCN2, and mTORC1 was examined. The results showed that serine supplementation alleviated the increased expression of Caspase 3 and GCN2 (Figures 6A,B,D), while improving the decreased expression of PCNA and mTORC1 expression in DSS-induced mice (Figures 6A,C,E).

## Discussion

Dextran sulfate sodium induction is the most widely used experimental model for UC. In these animal models, DAI value and colonic weight and length are critical markers for evaluating colitis severity (Osada et al., 2008). In the present study, DSS induced a significantly higher DAI (weight loss, gross rectal bleeding, and severe diarrhea) in mice. Moreover, in addition to decreased colonic weight and length, the colonic length and weight ratio was also decreased. These results indicate that DSS treatment results in overt features of colitis. Recently, L-serine has been generally considered as safe by the United States Food and Drug Administration since patients did not show any side effects in phase II clinical trials (Metcalf et al., 2018). In addition, our previous studies showed that mice supplemented with 1% serine in the drinking water did not showed any toxic effects on liver and small intestine (Zhou et al., 2017a,b). These results suggested that administration of serine is safe when it is used in mice. Although no previous reports have indicated that L-serine has any effects on UC, our previous study demonstrated an anti-inflammatory effect on LPS-treated mice. Thus, as expected, we found that the DSS-induced changes were reversed following L-serine supplementation for 7 days.

Dextran sulfate sodium directly targets the intestinal mucosa, damaging the intestinal epithelial cells of the basal crypts and impairing mucosal barrier integrity (Wirtz and Neurath, 2007). Moreover, recent studies have highlighted that remarkable changes in gut microbiota composition trigger intestinal inflammation in DSS-induced colitis (Hakansson et al., 2015; Munyaka et al., 2016). Therefore, we investigated the effects of serine on histopathological changes in the colon and gut microbiota dysbiosis following DSS treatment; our results demonstrated that serine improved intestinal integrity and gut microbiota composition in DSS-induced colitis. In addition, the biofunction prediction of microbial communities further demonstrated DSS-induced functional alteration in colonic microbiota and that the bacterial functional activities or metabolic pathways in serine-treated mice are more similar to those in control mice than in DSS-induced mice.

It has been widely reported that bacterial species diversity is reduced in both fecal and intestinal mucosa-associated microbiota samples from human patients or animal models with UC (Manichanh et al., 2006; Andoh et al., 2007; Samanta et al., 2012). However, a study using a pool of colonic and cecal content showed no significant difference in α-diversity analysis (Berry et al., 2012). Our results are consistent with this study as the results showed that DSS only tended to decrease intestinal microbial community evenness (Shannon H). These inconsistences suggest that the effects of DSS on microbiota composition may vary according to the anatomical site from which the sample is collected or whether the samples are from mucosa or feces. Nevertheless, a shift in bacterial community composition was confirmed in all these previous studies, as well as in the present study. In addition to reduced bacterial diversity, an increase in the *Bacteroidetes* to *Firmicutes* ratio also constitutes a significant characteristic of UC (Wang et al., 2017). Our results for samples from DSS-treated mice were consistent with this alteration (a reduction in *Clostridia* at the class level and in *Firmicutes* at the phylum level), while serine supplementation alleviated these changes. These results suggest that serine helps reverse the DSS-induced decrease in microbial evenness and community composition changes.

Prediction of metagenome functional composition showed that there was a difference in major perturbation in all three treatments. Alterations in amino acid metabolism were previously reported in UC patients (Morgan et al., 2012). In the present study, serine supplementation reversed the increased abundance of amino acid metabolism genes and decreased the abundance of nucleotide metabolism genes in DSS-treated mice. Serine not only links biosynthetic flux from glycolysis to purine synthesis (Parker and Metallo, 2016), but is also the major substrate for either glycine or pyruvate synthesis (El-Hattab, 2016). These critical steps of intermediary metabolism may be the reason that serine helps restore glycolysis; glycine, serine, and threonine metabolism; and pyruvate and purine metabolism to close to normal levels. In addition, alteration of microbiome amino acid metabolism may further affect amino acid requirements in colonic tissue. The increased expression of GCN2 and the decreased expression of mTORC1 suggest a lack of amino acids in the colonic tissue of DSS-treated mice. Interestingly, serine supplementation also alleviated these changes in colonic tissue in addition to its beneficial effects on amino acid metabolism in microbiota. A decrease in purine biosynthesis modules was reported in UC, consistent with our observations (Morgan et al., 2012), while serine supplementation alleviated this decrease. Serine is the major source of 1-C units for *de novo* synthesis of purine nucleotides and deoxythymidine monophosphate (de Koning et al., 2003). These substrates, as well as serine metabolism products, are essential for cell proliferation. The colonic microbiota of serine-supplemented mice was associated with increased DNA repair and recombination proteins and DNA replication proteins, leading us to further explore the effects of serine on cellular DNA replication and repair and proliferation (PCNA) and apoptosis (Caspase 3) markers in colonic tissue. Unlike amino acid metabolism, the replication and repair and cell growth and death functions in the microbiome were consistent with decreased apoptosis and increased proliferation in colonic tissue. These results indicated that the increased DNA repair and replication proteins by the colonic microbiota of serine-supplemented mice, according to the prediction of metagenome functional composition, had beneficial effects on the renewal of colonic tissue.

Relapsing episodes of UC are related to over-production of proinflammatory cytokines, which further impair intestinal permeability and cause severe colonic infiltration and dysfunctional gut barrier. Impaired gut epithelial barrier function may lead to the augment of inflammatory responses and permeability (Hering and Schulzke, 2009). Following serine supplementation, the increased concentrations of proinflammatory cytokines, MPO and EPO (indicators of colonic infiltration with polymorphonuclear leukocytes and eosinophils, respectively) (Han et al., 2015; Zhang et al., 2015) in the colon tissue of DSS-treated mice were reversed. These results indirectly indicate that serine supplementation help the recovery of intestinal integrity and barrier function, although we did not detect the expression of tight junction proteins which can directly reflect gut barrier function. In addition, the recovery of colonic morphology and the decreased apoptotic rate also indicated that the colonic dysfunction may alleviate by serine supplementation in DSS-treated mice.

## Conclusion

Our results show that serine supplementation reversed DSS-induced colitis in mice. To the best of our knowledge this is the first study to demonstrate that serine supplementation alters the composition of the colonic microbiota, as well as their functional profiles in colitis mice. Importantly, the effects of serine on the recovery of major perturbations of microbiome functions, such as glycine, serine, and threonine metabolism; replication and repair; and cell growth and death, may contribute to the renewal of colonic tissue in DSS-induced colitis. These results support the therapeutic potential of serine for regulating dysbiosis in UC. However, we unfortunately did not have a serine-only treated group since our results showed the unexpected and remarkable effects of serine on the microbiota composition in DSS-induced colitis mice in the present study. Because these results indicated that serine may be important for the composition of microbiota and their metabolism in the intestine, future works are suggested to explore the effects of dietary serine supplementation or serine deficiency on microbiota composition. In addition, future works are also needed to fully elucidate the mechanisms related to the effects of serine on colitis and to explore the beneficial effects of serine on other mouse model of colitis.

## Author Contributions

XhZ and HZ conceived and designed the research and critically revised the manuscript. HZ, RH, and BZ drafted the protocol. HZ, XhZ, HY, and XmZ contributed to the literature search, interpretation, writing, and proofreading of the manuscript.

## Conflict of Interest Statement

The authors declare that the research was conducted in the absence of any commercial or financial relationships that could be construed as a potential conflict of interest.

## Footnotes

1 https://www.cusabio.com/
